# Training Prescription and Monitoring in Rowing: Perspectives From Elite Australian Coaches

**DOI:** 10.1002/ejsc.12328

**Published:** 2025-06-07

**Authors:** Sophie P. Watts, Martyn J. Binnie, Paul S. R. Goods, Peter Peeling

**Affiliations:** ^1^ School of Human Sciences (Exercise and Sport Science) The University of Western Australia Crawley Australia; ^2^ Western Australian Institute of Sport Mount Claremont Australia; ^3^ PHysical Activity Sport and Exercise (PHASE) Research Group School of Allied Health (Exercise Science) Murdoch University Perth Australia; ^4^ Centre for Healthy Ageing Health Futures Institute Murdoch University Perth Australia

**Keywords:** athlete preparation, coaching, performance assessment, rowers

## Abstract

Elite coach experiential knowledge may provide valuable insights into athlete preparation and monitoring practices that are otherwise difficult to establish objectively. Accordingly, we explored the perspectives of elite Australian rowing coaches in relation to (1) training philosophy, (2) training prescription methods, and (3) training monitoring and performance assessment. Ten experienced rowing coaches (experience range 15–51 years) were interviewed in a semi‐structured format on the three topics established above. Data were analysed using hierarchical content analysis to develop higher and lower order themes. Under training philosophy, three higher order themes were established: (1) building the engine, (2) intensity is periodised and polarised, and (3) progression of on‐water speed is key. Two higher order themes were established for training prescription methods: (1) prescription is modality dependent and (2) athlete characteristics are important. Under training monitoring and performance assessment, two higher order themes were identified: (1) assessing on‐water performance is difficult and (2) short versus long term monitoring. Coaches acknowledged the complexity of training prescription and quantification in rowing and offered practical methods to overcome challenges with these processes. These findings help to inform coaches and support staff in the training environment as well as inform future research.

**Summary**

- Training prescription and performance monitoring in rowing is complex, largely due to the impact weather conditions have on these practices.
- Coaches may rely on athlete self‐regulation for training prescription and relative performance to assess progression in a real‐world setting to overcome challenges.
- Improvements in the practical use of boat instrumentation for the measurement of on‐water power may provide a more holistic method of athlete preparation and monitoring.

## 1 Introduction

Coaches' play an integral role in the physical and technical preparation of rowing athletes through training prescription and monitoring practices; however, there is limited research exploring coaches' perspectives on these practices (Timmerman et al. 2024). An increased understanding of what coaches deem important and practical in a high‐performance rowing environment will enhance training and highlight relevant areas for future research.

To date, research has primarily focussed on quantitative methods to understand the physical and technical demands of rowing performance (Holt et al. 2020; Otter‐Kaufmann et al. 2020). Training is typically described according to a three‐zone intensity distribution model (Z1: intensity below first lactate threshold, LT1; Z2: intensity between LT1 and the second lactate threshold, LT2; Z3: intensity above LT2), and is reported to follow a pyramidal (Z1 > Z2 > Z3) or polarised (Z1 > Z3 > Z2) training intensity distribution (TID). This training organisation, characterised by a high volume of low‐intensity (Z1) (Plews et al. 2014; Tran et al. 2015), is thought to allow for aerobic energy system development and technical/skill reinforcement. Additionally, bouts of training above LT2 (Z3) provide a high‐intensity training stimulus for physiological adaptation (Driller et al. 2009; Ní Chéilleachair et al. 2017), allowing athletes to practice the technical components of ‘race‐pace’ rowing. However, research has neglected to consider the experience‐guided perspectives of rowing coaches, with a focus on how they design and implement training programmes to maximise both physiological and technical adaptation.

Alongside optimal training prescription, monitoring of rowing training and performance is important to ensure progressive overload is maintained while non‐functional overreaching avoided. However, monitoring on‐water training and performance can be complex (Binnie et al. 2023), primarily due to the impact of the environment (wind speed and direction, water temperature, flow) on boat speed (Kleshnev 2009). Accordingly, rowing ergometers are commonly used to assess athlete physiology and performance, as they simulate the physiological demands of on‐water rowing (Lamb 1989; Mäestu et al. 2005), and can be performed in a controlled environment. Additionally, boat instrumentation systems that monitor power at the oar, or geospatial coordinates of the boat, may be used to aid in the technical assessment of on‐water rowing performance (Holt et al. 2021). However, these devices are expensive, and their analysis is time‐consuming. As such, an understanding of the coaches' considerations for monitoring athlete training and performance may help bridge the gap between science and practice.

Elite coach knowledge is gained through years of experience in a high‐performance environment (Greenwood et al. 2012), and provides valuable insights that are otherwise difficult to establish objectively. Experiential coaching knowledge has added depth and understanding to the transfer of resistance training in elite cycling (Burnie et al. 2018), and movement in tennis (Giles et al. 2019). In rowing, coaches' perspectives have been examined in relation to performance, talent and progression (Legge et al. 2023), and technical evaluation on‐water (Baumann and Schmid 2024). From these studies, Legge et al. (2023) provided valuable insights on how to develop junior rowers while Baumann and Schmid (2024) established some subjective technique criteria which may be helpful for upskilling the novice rowing coach. However, given the complex interaction of physical and technical variables in rowing, as well as the difficulty in quantifying and monitoring performance, there remains scope to explore coaches' training philosophies and their perspectives on prescription and monitoring practices.

Therefore, the aim of this investigation was to use a qualitative approach to explore the experiences and perspectives of high‐performance rowing coaches in three main areas: (1) training philosophy, (2) training prescription methods, and (3) training monitoring and performance assessment.

## 2 Material and Methods

A small *q* qualitative approach with a post‐positivist perspective was adopted for this cross‐sectional study (Clarke and Braun 2021).

### 2.1 Participants

Ten rowing coaches (8 male, 2 female) with > 5 years' experience (mean experience 27 ± 12 years, range 15–51 years) were recruited for this study. All coaches were currently coaching within the Australian high‐performance system and were currently, or had previously, worked with underage or senior elite international‐level athletes (McKay et al. 2022). Signed informed consent was obtained from all coaches. This study was approved by the Human Research Ethics Committee of the Host Institution (2023/ET000842).

### 2.2 Data Generation

An interview guide was developed, covering three main topics: (1) training philosophy, (2) training prescription methods, and (3) training monitoring and performance assessment. A pilot interview with a state institute coach, not involved in the study, was conducted to refine the interview guide. Coaches were subsequently recruited to undertake a verbal, one‐on‐one, semi‐structured online interview. The lead investigator (S.P.W) conducted all interviews, which averaged 55 ± 10 min, and were recorded via Microsoft Teams (Microsoft Corporation, Washington, US).

All interviews were transcribed verbatim by transcription software (Microsoft Teams, Microsoft Corporation, Washington, US). Transcripts were reviewed for accuracy by the lead investigator and then sent to the coach to ensure their views were accurately reflected or to make amendments they deemed appropriate. Data (transcripts) were then imported into a qualitative analysis software (NVivo20, QSR International, Melbourne, Australia).

### 2.3 Analysis

Hierarchical content analysis was used to develop higher and lower order themes (Sparkes and Smith 2013). The lead researcher immersed themselves in the data by reading and listening to the transcripts and interviews multiple times. Data were coded, primarily at the semantic (explicit meaning) level (Braun and Clarke 2022), and an inductive approach was employed to emphasise data‐based meaning from participants (Byrne 2022). However, a degree of deductive analysis was employed to ensure that open‐coding contributed to producing themes relevant to the research question (Byrne 2022). Codes were clustered into meaningful categories to create higher and lower order themes (Sparkes and Smith 2013). The codes and themes (higher and lower order) were then cross‐checked against the data to ensure appropriate exploration, identification, and representation of relevant information. A ‘critical friends’ process was adopted to ensure rigour, improve the quality of analysis, and explore multiple and alternate explanations and interpretations of the data and themes (B. Smith and McGannon 2018). During this process, the authors engaged with the lead investigator on quotes and raw themes, providing constructive critique so that themes were refined.

## 3 Results

During transcript analysis, the following higher order themes were generated. For **training philosophy**, codes clustered into three higher order themes: (1) ***building the engine;*** (2) ***intensity is periodised and polarised;*** (3**) *progressing on‐water speed is key***. For **training prescription methods**, two higher order themes were established: (1) ***prescription is modality dependent*** and (2) ***athlete characteristics are important***. Within **training monitoring and performance assessment**, two higher order themes were developed (1) ***on‐water performance is difficult to quantify*** and (2) ***short*** versus ***long term monitoring***. Higher‐ and lower‐order themes are discussed for each topic area below. Quotes are provided throughout the Discussion and in Table 1 (**training philosophy**), Table 2 (**training prescription methods**) and Table 3 (**training monitoring and performance assessment**).

**TABLE 1:** Training philosophy—summary of higher and lower order themes, and quotes.

Higher order theme	Lower order theme	Quotes
1. Building the engine	a. Volume is the goal, but consistency of training is key (*n* = 6)	C5: So I guess my general philosophy is volume is the overarching principle around whatever I'm doing. So in the prep phase, it's definitely volume…So then consistency of volume from that period all the way through…. Consistency of training is probably one of the biggest ones, and that then has a lot of other flow on
		C9: The main one is their ability to maintain training loads over time…. Consistency of training overtime. …By far the number one marker of anything
	b. Robustness needs to be developed to handle training load (*n* = 8)	C5: …probably on the same sort of spectrum as the strength component and robustness, so are you stable enough, do you have the right flexibility etcetera to get yourself in the right position over and over again?
		C4: Third thing is making sure they're robust enough, so enough strength and conditioning, ab strength, lower back strength and the kinetic chain from the back to the hamstring through the glute
2. Intensity is periodised and polarised	a. Intensity becomes more polarised as racing approaches (*n* = 10)	C9: … if you're winding the clock on you've got more intensity coming into the programme and your volume is coming down
		C10: And with the intensity, we're obviously just trying to edge up towards that top rate speed and that racing intenseness that we need going into sort of February through to April
	b. Recovery between ‘high’ intensity sessions is important (*n* = 8)	C6: I'll try and make it minimum of 48 h. But, normally it would be on a maybe on a Thursday, Saturday or it might be that Tuesday, Saturday even. But either of those would be fine as long as it's, I think generally 48 h
		C3: …If we're on hard or heavy week, then we try within the heavy week to sort of medium‐heavy‐medium, to give some recovery time. So we will volume row, intensity session, volume row, intensity session across all week, in a bit of a wave format
3. Progressing on‐water speed is key	a. Ability to hit and hold prognostic speed is indicative of performance (*n* = 6)	C8: …they might be doing a session where they have to sit on race speed, how long can they sit on race speed for? And then that's a good indicator. The longer they can stay there, that's a really good indicator of how they're gonna perform as well
		C10: So the intensity of what they do steps up like once they've got that base and once they've got that technical stability then we try and get their intensity up. So they're actually holding a pace consistently for 18–20 K, being able to technically hold that as well all the way through

**TABLE 2:** Training prescription methods—summary of higher and lower order themes, and quotes.

Higher order theme	Lower order theme	Quotes
1. Prescription is modality dependent	a. Ergo is about power, on‐water is technical and physical (*n* = 6)	C7: So we outline, I guess, what are the parameters that we want to work inside? We say, OK, we want to be working at this sort of intensity internally. You know, your heart rate should be in this band. We want our stroke rate to be 18 to 20 and we want the boat speed to be in this range if it's going well. And so if anything's not inside any of those boxes, then OK, we've gotta work out why and try and fix the problem
		C6: So if for example if we wanted to continue to really reinforce the athlete, the boats to row really good distance per stroke. So they're rowing, practising rowing really hard, pushing the boat, rowing really long and having a really high contrast in their drive to recovery rhythm. …We might spend a significant portion of the session at rate 16 or 16/18/20 and have some graduations there, but keep bringing them back down to 16, which allows, it can still be an incredibly hard session, but it'll keep the heart rate zones a little bit lower
		C4: So generally on the ergo it's wattage and stroke rate
	b. Weather conditions can impact prescription, particularly speed (*n* = 7)	C1: …it would only change whether I can give them a target or not [speed target, Authors input]. So, if I can't give them a target, I'll give them an RPE [rating of perceived exertion, Authors input] … You can give them rate targets, that's fine weather doesn't affect that. But speed targets, yeah, will change, and are very hard to predict
		C10: We're pretty windy here at West lakes …I mean sometimes we'll change if it's super windy, we'll come in and do alternate training on the bike or on the ergs. But we do tend to row in most conditions here because we think it just prepares the athletes for future… Sometimes you just go on the heart rate rather than the boat speed depending on like the conditions
2. Athlete characteristics are important	a. Reliance on athletes developing self‐awareness (*n* = 7)	C3: … I use my 2 benchmark pairs to effectively find their T2 rate. I will tell them that it's rate 20 at T2 heart rate and they know what the pressure is from that
		C5: … the athletes have got a really good regulator in terms of how hard they're working. So they'll stick with the rate, distance per stroke, speed is a bit variable, but then how hard they're working, their internal regulator is pretty accurate as well
	b. Understanding the athlete is important (*n* = 7), but individualising can be difficult (*n* = 3)	C6: And I think getting to know the athletes and what makes them tick and then also what are the their areas of opportunity is really important because all of those things are important, like at the end of the day the person that will win the race is the one that … they've you know got that balance that the best out of everybody that they're racing at that point in time. And for some athletes there's a bigger contribution from a particular area than another
		C3: So it's not a one size fits all things. So there are some people where their mid race for a whole variety of reasons might be only sort of early 30s [SR, Author input] and there are others where their mid race could be you know late 30s. So, when you start to get into these sorts of patches, say with set rates… one of them is going to be overextended and others going to be within that. So, there's a bit of bespoke stuff that sort of goes on as you look at the pairs
		C5: And then I think it's always just having the time to individualise and analyse individual programmes and also how they're adapting and recovering. So typically, we'll have a generic programme, you modify maybe on the erg or something, getting a bit more layers and depth around how you can individualise training programmes based on their physiology, their fatigue etc. and their life balance, I think is the other part that we just don't have time to probably do the depth we could

**TABLE 3:** Training monitoring and performance assessment—summary of higher and lower order themes, and quotes.

Higher order theme	Lower order theme	Quotes
1. On‐water performance is difficult to quantify	a. Due to the impact of weather, relative performance becomes important (*n* = 10)	C6: …your best comparisons in terms of like say longitudinally or even just session to session are the crews I think, so prognostic data. So having some sort of standard performing boat or boats in your group that you have an understanding of their competitiveness, let's say. And then comparing the rest of your crews to those boats
		C8: … we will look at the prognostics of that so that they can be compared. So who's where? …the conditions can be completely different, but at least this person's consistently #1 prognostically…I'd say we're looking at that prognostically where they are ranking wise…and the monitoring sessions where they sit prognostically relative to other people
	b. SR‐speed relationship is valued (*n* = 9)	C2: So boat speed primarily and boat speed to rate relationship I value really highly. I do look at you know speed for distance as well, so if we're doing a 5 K time trial versus a 1500 m piece, obviously their ability to be able to hold better speeds even at the same rate is going to be more easily achieved at the shorter distances. So I find that relationship interesting and then being able to compare that to different athletes too
		C4: …If the speed is coming up relative to their rate…We watched people's trend lines in how they're performing and in theory they should be able to rate 28 and do 90% [prognostic speed, Author input] for step rate. If they can do that they're probably on track
	c. Data accuracy/technology is lacking (*n* = 9)	C3: I'd love to get catch consistently; I'd like to look at slip. I'd like to look at ark. I really would like to know effective length measures more regularly than I get to. I'd like definitely to be able to know within a session what the watt measure is. But I can't do that unless I'm biomeched up and I've got the stuff in the boat which yeah I don't have
		C6: Really one of the ones that's still the hardest to do is to get real time speed feedback, we have some systems that do it, but they're unreliable and not real time enough, not accurate enough, like really be able to see what is happening with the boat right in this moment now
2. Short versus long term monitoring	a. Physiology is assessed by ergo testing and monitoring (*n* = 10)	C5: Obviously 2 K test, 5 K max tests, from pure performance point of view on the ego….. But RPE and lactate are probably the other ones that we track pretty heavily on the ergo…..[the 7 × 4 min graded exercise test, Authors input] So I use it A. to determine their aerobic and anaerobic contribution. So get an understanding of do they need more work in any particular area. So what their physiology looks like, how much lactic they're producing, what their raw score is, but also what that shift in lactate curve looks like. So are they adapting to training overtime, can they produce more power for less lactic, classic sort of physiology stuff. And then the raw 4‐min time or distance they've done at the end of it, can they produce more power? Can they row longer? So looking at improvements in physiology from a lactate curve shift point of view, change in their actual aerobic contribution to training, and then absolute power they're producing
		C6: I think 2 km ergo score is still a really effective and really key performance indicator. It requires not only the physiological and the physical capabilities and technical but also the psychological capabilities because they're painful and so they're a great indication of what an athlete is potentially capable of……. probably the most consistent piece of measured work that we've done through this is just a simple 2 by 30 min ergo at rate 18 and we're just looking at their ability to overtime produce more power for the rate and for the same RPE and lactate and it can, there's been there's some big shifts over that period of time as you say longitudinally
	b. Global trends of volume and intensity are helpful for load management and fitness tracking. (*n* = 7)	C1: And for load tracking, it's more about just seeing global loads as a usable number that can help us make a decision if they've done too much load or too little load. So it just a convenient metric to be able to track kilometres and hours because our programme is so similar week in, week out. The intensity band, we will look at the heart rate intensity bands to see if people are maybe over working or we're doing something wrong. But the assumption is that programmes pretty similar week in, week out. So if we just look at how much of that is completed, we'll get a relatively good picture with the most simple data
		C9: I tend to think that daily monitoring in terms of something like using training peaks and plotting it over time is equally as beneficial as the formal tests that we will actually do

## 4 Discussion

### 4.1 Training Philosophy

A key theme developed from discussing the coaches' **training philosophy** was ***building the engine*** of the athlete. A high volume of training was discussed by all coaches, however, a subset of coaches also agreed that training consistency is key to influencing athlete development and should be considered in programme design. As explained by C4, a senior national team coach with 23 years experience:

Alright, well I suppose the first thought is that consistency and continuity is the number one thing. So, writing a programme or managing an athlete in the programme that allows them to complete the programme…if they can do more training and get more of it completed, they're probably going to be better athletes by the end of it.

Injury and illness data over an 8‐year period in international‐level rowers showed overuse injuries were more frequent than acute injuries and illness represented 32% of all causes of lost training time (Trease et al. (2020)). Loss of training time due to injury or illness has been reported as detrimental to performance in other sports (Hägglund et al. 2013; Podlog et al. 2015; Raysmith and Drew 2016). Therefore, implementing training programmes that allow athletes to consistently train is perhaps more important in the coach's perspective than completing specific volume and intensity targets.

Moreover, coaches identified the importance of athletes developing strength and robustness to handle high training loads. The terms ‘core,’ ‘flexibility’ ‘strength’ and ‘strength endurance’ as well as major muscle groups such as the ‘glutes’, ‘hamstrings’ and ‘lower back’ were used by coaches to convey the idea that athletes needed to be ‘well‐rounded’ so they can repeatedly execute the rowing stroke. As participant C9, an Olympic medal winning coach with 51 years experience, explained:

Certainly, in the early part of the season they’ve got to get stronger…., they've got to build robust bodies… so you can use the word core all you like. But it's true, they need to develop that, they need to develop connective tissues more and that's things like hamstrings and glutes and ligaments, tendons, so they need a bit of all roundness.

Strength and power have been linked to metrics associated with on‐water rowing performance (Legge et al. 2024), with movement competency (e.g., flexibility, trunk musculature) highlighted by coaches as a key difference between junior and senior rowers (Legge et al. 2023). Additionally, the development and maintenance of trunk strength and endurance may contribute to reduced lower back pain in rowers, thus decreasing the training time lost to injury (Nugent et al. 2021). As such, developing strength and robustness likely plays a role in improving rower performance, whilst helping maintain training consistency by reducing the likelihood of injury.

The second theme identified around **training philosophy** was the ***periodisation and polarisation of intensity***. Here, all coaches conveyed that most training time (∼80%) was performed at a low intensity (below LT1), with relatively small proportions of time spent at ‘threshold’ or above (>LT2). This supports current literature describing the training intensity distribution of elite rowers, where 77%–83% of training time was spent in Z1, 15%–17% in Z2, and 1%–6% in Z3 (Plews et al. 2014; Tran et al. 2015). However, coaches highlighted a shift in the distribution of intensity as athletes progress towards racing, with more sessions targeted specifically at ‘race‐pace’ (i.e., training becomes more polarised) during this time. As C7, a senior national coach with 23 years experience, explained:

The general prep, I definitely have a predisposition towards a high volume, polarised type training. You know, large volume at low intensity, a relatively small amount of work at higher intensities…and then coming into a competition is where we would pull back volume to some degree… and start to layer in some work at or above race intensity to prepare for the competition…. and probably polarise even more like pull back the intensity at the low intensities and try to push the high intensity work to a really high level.

This intensity shift was noted by Arne et al. (2009), who investigated the training of young world‐class rowers. Here, it was determined that high‐intensity sessions shifted from ‘lactate threshold’ (Z2) towards ‘race‐pace’ training near VO_2max_ intensity (Z3) during the competition phase. Additionally, Filipas et al. (2021) compared the effects of pyramidal (Z1 > Z2 > Z3) and polarised (Z1 > Z3 > Z2) TID in endurance runners, reporting that moving from pyramidal to polarised training improved 5 km time‐trial performance compared to other forms of periodisation. As such, rowing coaches' experiential knowledge of training periodisation appears to be consistent with current quantitative research findings. Likewise, weekly training macrocycles appeared to follow a similar distribution, whereby high‐intensity sessions were separated by 24–48 h and interspersed with low‐intensity sessions. As C1, a state institute coach with 18 years experience, described:

We try and space them [high intensity training sessions, Authors input] as evenly through the week as possible to achieve maximum intensity and also recovery. So, Tuesday morning, Thursday afternoon, Saturday morning, it's sort of as evenly spaced between the week as we can get.

Research in rowing suggests that greater time spent training above 80% HR_max_ may be associated with extended parasympathetic suppression, and thus limits the number of high intensity sessions that can be performed in a training week (Holt et al. 2019). As such, training polarisation and allowing sufficient recovery between high‐intensity sessions may help avoid non‐functional overreaching.

The final higher order theme under the topic of **training philosophy** was ***progressing on‐water speed is key***. A subset of coaches highlighted the ability of athletes to hold/maintain consistent boat speed over time as a key indicator that physical and technical elements are aligned in the boat, and that athletes are progressing. As C5, an underage national team coach with 23 years experience, summarised:

…over time you're trying to make sure that you're achieving that boat speed for as long as possible. So, you might have a target, but can do it for 20 min, but it's still a target you're trying to reach for two hours or whatever it is.

It is common practice in rowing for the world's best time (WBT) of each boat class to provide a fixed benchmark for indexing speed, often termed ‘prognostic’ or ‘gold standard’ speed (Kleshnev 2020). Given the well‐established relationship between SR and boat speed (Holt et al. 2022; Kleshnev 2020), submaximal SRs are often prescribed to a scaled prognostic boat speed as a way of informing training standards. As an example, in the Australian high‐performance rowing system the goal is to hit 80% prognostic speed at 20 spm (Binnie et al. 2023). However, given most WBT are set in the best‐case scenarios of athlete performance (world‐class field, tapered) and environmental conditions (warm water, tail wind, minimal flow), the use of these standards in a training environment with considerable variability (athlete and weather) has limitations.

To summarise training philosophy, coaches' description of training volume and intensity was consistent with current literature for endurance training, whereby high volumes of low‐intensity training are completed, interspersed with periodised high‐intensity training sessions that increase in frequency towards competition. Additionally, training consistency and the development of strong and robust bodies were identified as crucial for training progression and improvement of on‐water rowing speed.

### 4.2 Training Prescription Methods

Two higher order themes were established under training prescription methods. The first was that ***training prescription is dependent on the modality of the session***. Quantitative research describing the training of rowing athletes notes that various exercise modalities (rowing, running, cycling, strength) contribute to overall training time, although rowing modalities (on‐water and ergometer) are dominant (Tran et al. 2015; Treff et al. 2021). Ergometer rowing may elicit similar physiological demands to on‐water rowing (Vogler et al. 2010) and allows training to occur in a controlled environment. Coaches referred to the use of power or pace as prescription variables for ergometer sessions to ensure an appropriate physiological stimulus. However, significant differences in arm motion (Lamb 1989), handle velocity, and acceleration profiles (Kleshnev 2005) are reported between stationary ergometers and on‐water rowing. Although there is evidence to suggest a dynamic rowing ergometer better simulates the technical demands of on‐water rowing (Elliott et al. 2002), the Australian system utilises a stationary ergometer for all testing. Regardless, given the complexity of rowing technique, the specificity of on‐water training is important for skill development. When discussing on‐water training prescription, coaches considered the context of both physiological and technical stimulus, with SR often used as a variable to dictate these. As C2, a state institute coach with 19 years experience, summarised:

…there's probably a 50‐50 split for the ergos between done to time versus done to distance and, I'm lucky enough that they have training zones, that they've all been tested in the lab. So, they've got target wattages and numbers that they're trying to hit. And we'll just flag whether it's an all‐out effort, let's see what you can do or what part of that session might be all out versus just hit your numbers, do the session well, concentrate on the quality of your movement pattern, concentrate on the quality of your rhythm and those kind of things…

[Paraphrased for clarity] For on‐water training, I set a specific distance for the session, but I also pay attention to how long it takes to cover it. I give my athletes a target stroke rate—strokes per minute. By combining that with the distance and the heart rate intensity were looking for, this helps make sure we’re hitting the right training zone and building both physical fitness and technical skills.

Coaches further commented on the complexity of on‐water training prescription by highlighting the impact of various weather conditions (wind, water temperature, flow), particularly when prescribing by boat speed. As C3, a club and state institute coach with 43 years experience, explained:

…the training environment that we train in, we're on a river that's heavy tidal. Where half our session is going to be against it, half our sessions going to be with it every week. And it's going to be different each week in that some weeks you're going to be with it for the early part and against it coming home and then other weeks that's flipped on its head. And the difference because of the tidal flow of the river that we're on can be significant. So, the first thing is I can't prescribe anything based on standard split speed.

As mentioned previously, training based on boat speed (prognostic speed) was valued by coaches, however, the ability to prescribe training standards based on speed is limited in a real‐world scenario where environmental conditions are ever‐changing. This flows to the second theme for **training prescription methods**, which was that ***athlete characteristics are important***. Coaches discussed the benefits of athletes developing self‐awareness and self‐regulation with regards to their intent in a session. The use of self‐regulatory skills such as reflection, planning, self‐monitoring and evaluation are important for learning (McCardle et al. 2019) and are beneficial for sport performance (Jonker et al. 2012). As C6, a senior national team coach with 20 years experience described:

…I think it's actually really important for them to have a more familiar relationship with the energy output that they need to achieve the gold medal speed. So, on good condition days they understand what it costs to be able to sit on those speeds. So that when the wind is not great, they're more in tune with being able to deliver the same effort and then learn what the impact on their speed is in those conditions. And then gradually over time they become more educated, they should be able to anticipate a little bit more, this sort of 1–2 m per second head breeze is going to slow the boat down by 4–5 seconds per 500. And so then with their knowledge and their awareness of their energy expenditure combined with the boat speed and wind conditions allows them to make a determination as to whether they’re training at the right level.

Furthermore, C7 explained how understanding the athlete is an important aspect of being able to optimise training for the individual.

Because ultimately what's important is the 2K performance on the water. And you might have to understand how do different individuals create that performance. You can have people that paddle really slowly, that race really, really fast because of how they generate their power, what they bring physiologically to a 2K performance. They're stroke length, some efficiencies, different things that change between low and high intensity. So, you use that information to try to #1 improve them, and also to understand how people create the performance, and then you can help them to train more effectively for them.

However, there was a small number of coaches who expressed that training is difficult to individualise when working with a large group of athletes; C4 explained:

The harder thing is actually in group training prescription, individualising so the athlete gets the benefit they need. That is the tricky part of a group squad training… is getting the prescription right for the group and the individual in the group.

To summarise, rowing training prescription is complex and dependent on the modality of exercise performed. Ergometer training tended to have a physical focus, whereas on‐water training encompassed both technical and physical stimuli, with environmental conditions impacting prescription and further adding complexity. Accordingly, coaches may rely on athlete self‐awareness and self‐regulation to increase training prescription adherence.

### 4.3 Training Monitoring and Performance Assessment

In the third topic area, **training monitoring and performance assessment**, there were two key themes developed. Firstly, coaches expressed that ***on‐water performance is difficult to quantify***, which supports the general perspectives of recent commentary (Binnie et al. 2023). Such difficulty is likely due to many factors, but the impact of environmental factors on boat speed (wind speed and direction, water temperature, flow) appears key, meaning that relative performance (i.e., how athletes are performing compared to each other) becomes important in assessing on‐water outcomes. As C10, a state institute coach with 15 years experience, explained:

…our senior athletes will be pretty consistent, not dependent on the weather… so we've got a couple of really experienced athletes in our DTE [daily training environment, Authors input] and so what I'll look at is where they're sitting and then where the younger athletes are sitting compared to them.

Given the high training demands of rowing, maximal efforts at sub‐race distances or prolonged efforts at sub‐maximal intensity are commonly used as proxy measures of performance at various stages of the season (Binnie et al. 2023; T. B. Smith and Hopkins 2012). As such, using prognostic speeds and scaling athletes in these sessions may help inform athlete performance and progression in context of environmental conditions. However, relative performance is not without limitation, as it relies on the quality and consistency of athletes within the training environment.

While acknowledging the influence of environmental conditions on boat speed, coaches also highlighted the value of the SR‐speed relationship to assess performance and progression; C7 explained:

…we'll have speed targets as the rating goes up all the way to understand what's the relationship between rate and speed, between rate 18–20 at the bottom and rate 40 when we're at the top. You want to be working along that continuum pretty well all the way. And, if you're not able to progress the speed and the rate together, well then that's a sign of what you have to then work on in the low intensity work, either technically or physically to achieve that.

Stroke rate is one of the key determinants of rowing performance, albeit limited by mechanical conditions (inertial losses of energy increase with SR) and the neuromuscular abilities of rowers (higher SR requires faster muscle contraction, and quicker coordination of movement) (Kleshnev 2020). Additionally, rowing biomechanics and technique are different at low and high SRs, with higher SRs having shorter drive and recovery times, faster movement velocities, and higher requirements for force and power (Kleshnev 2021). As such, understanding the relationship between SR and boat speed for an individual or a crew may provide insight into technical and physical deficits.

When coaches were asked what data they would capture if not faced with limitations (equipment cost, time cost), the metric of on‐water power was frequently discussed, while the need for improved data accuracy and better real‐time feedback on weather, technique and physiological data was also highlighted; C1 summarised,

…It's not so much we need more data or different data, we just need more assurance over the accuracy of the data on water. So, the accuracy of the weather measurements, the accuracy of the stroke rates that people are doing. And in an absolute perfect world, we'd also be looking at putting telemetry on boats and measuring wattage. So, actually measuring force per stroke over arc length and being able to report on that, that would be the ideal world.

Although attempts have been made to correct boat speed for environmental conditions (Diafas et al. 2006; Pomerantsev et al. 2022), there is no current standardised method of data collection or correction, and it is likely that individual training environments utilise their own bespoke systems (Binnie et al. 2023). Alternatively, on‐water power, measured via boat instrumentation, has a theoretical curvilinear relationship with boat speed (Hill and Fahrig 2009; Holt et al. 2022) and may offer a more direct assessment of intensity and boat performance despite being impacted the environment and athlete characteristics (size, technical efficiency). Research suggests that on‐water power has the capacity to predict 2000 m rowing speed (Holt et al. 2022), account for speed variances caused by real‐world environmental conditions (Hogan et al. 2022), and increase adherence to training intensity when used as an instantaneous feedback tool (Lintmeijer et al. 2019). Therefore, on‐water power may provide a more robust method of training prescription in addition to on‐water performance assessment in rowing; however, as reflected by coaches, boat instrumentation comes with significant financial cost and is likely impractical for everyday multi‐athlete use.

The second theme developed under **training monitoring and performance assessment** was ***short*** versus ***long term monitoring***, where all coaches discussed the use of ergometer testing to assess physiology and performance. National ergometer testing set out by Rowing Australia include 5000 and 2000 m time trials, as well as a 7 × 4 min graded exercise test for the assessment of VO_2max_ and lactate thresholds (Rice 2019a, 2019b). Additionally, coaches discussed the use of weekly ergometer training sessions to assess the physiological response (i.e., blood lactate and heart rate) to training; C2 explained:

Rowing Australia prescribes ergo testing probably once every sort of six weeks, so we're either doing a 5K or 2K ergo test which we can look into not just the physiological change, but what's the whole of system change in that athlete to produce that result. …Rowing Australia also prescribe 2 by 30 min ergos at T2 [intensity zone, Authors input] a week…that's one of the ones that we’ll often take blood lactate on as well to get an indication of, OK heart rate is the same, watts are the same but blood lactates gone down, terrific, seeing a physiological change… I think physiology is easier to measure on the ergo so that's where we do most of our stuff.

The complexity associated with quantifying on‐water rowing performance has led to the widespread use of rowing ergometers for performance assessment. The 2000 m ergometer time trial remains the most common measure of rowing performance (Mäestu et al. 2005). Additionally, the use of ergometer time‐trials and testing, combined with measures such as oxygen consumption, heart rate, or blood lactate, may provide important information on athletes' physiological status (Astridge et al. 2023). Therefore, scheduling ergometer testing and monitoring physiological metrics during ergometer training can help provide within‐season and between‐season performance tracking.

Finally, coaches discussed using long‐term tracking of global volume and intensity trends; C5 explained:

…so we'll have obviously a planned programme and then we'll track planned versus actual [training, Authors input]. And then definitely look at long‐term load management and then tapering, peaking, etc, where the threshold is in terms of if they get sick or injured… So typically, if you go back and use training peaks, looking at your TSB [training stress balance (form), Authors input] scores and training scores in there to monitor and track, so it's very much around how the individual is coping. But importantly, are they being overloaded appropriately and improving the physicality side.

This ties back to coaches valuing training consistency, whereby tracking planned versus completed training may help inform training compliance. Training quantification is common in endurance sports to evaluate an athlete's response to training loads, ensure adequate stress/recovery balance, and to determine the relationship between training and performance (Mujika 2017). Therefore, consistent long‐term training monitoring is important to support the programming and progression of rowing athletes.

To summarise, coaches agreed that on‐water performance was difficult to quantify, and as such, relative performance and ergometer time‐trials become important for performance and physiological assessment. Despite being impacted by weather conditions, speed and the SR‐speed relationship was highly valued by coaches, whereas the addition of on‐water power measurement was identified as a metric they would like to consistently capture if resources permitted.

## 5 Conclusion

We explored rowing coaches' perspectives on training philosophy, prescription methods, training monitoring, and performance assessment. On‐water speed was a valued metric for both prescribing and monitoring rowing training, despite being impacted by environmental conditions. Practically, utilising athlete self‐regulation and assessing relative performance on‐water were ways in which coaches overcame challenges associated with training prescription and performance monitoring. The findings from this study, however, are limited to a relatively small sample of coaches (*n* = 10) from the Australian High‐Performance rowing system and may not reflect the opinions of coaches in other Nations or at lower levels. Future research should consider broadening qualitative studies of rowing coaches to include multiple Nations, so that findings may be extrapolated to other parts of the world. Additionally, research into improving the practical use of boat instrumentation for the measurement of on‐water power may enhance current athlete preparation and monitoring practices in rowing.

## Ethics Statement

This research in Australia was approved by the Human Research Ethics Committee of the University of Western Australia (2023/ET000842), and written informed consent was obtained from each participant prior to undertaking the investigation.

## Conflicts of Interest

The authors declare no conflicts of interest.
